# A Survey on Biomimetic and Intelligent Algorithms with Applications

**DOI:** 10.3390/biomimetics9080453

**Published:** 2024-07-24

**Authors:** Hao Li, Bolin Liao, Jianfeng Li, Shuai Li

**Affiliations:** 1 College of Computer Science and Engineering, Jishou University, Jishou 416000, China; haoli@stu.jsu.edu.cn (H.L.); ljf@jsu.edu.cn (J.L.); 2 School of Communication and Electronic Engineering, Jishou University, Jishou 416000, China

**Keywords:** intelligence algorithm, optimization problem, zeroing neural network, bio-inspired algorithm

## Abstract

The question “How does it work” has motivated many scientists. Through the study of natural phenomena and behaviors, many intelligence algorithms have been proposed to solve various optimization problems. This paper aims to offer an informative guide for researchers who are interested in tackling optimization problems with intelligence algorithms. First, a special neural network was comprehensively discussed, and it was called a zeroing neural network (ZNN). It is especially intended for solving time-varying optimization problems, including origin, basic principles, operation mechanism, model variants, and applications. This paper presents a new classification method based on the performance index of ZNNs. Then, two classic bio-inspired algorithms, a genetic algorithm and a particle swarm algorithm, are outlined as representatives, including their origin, design process, basic principles, and applications. Finally, to emphasize the applicability of intelligence algorithms, three practical domains are introduced, including gene feature extraction, intelligence communication, and the image process.

## 1. Introduction

The question “How does it work?” has motivated many scientists. In past decades, biomimetics has promoted lots of science and engineering research and changed people’s lives dramatically. Neural networks, which are inspired by biological neurons, have achieved significant success and been used in impactful applications [1,2]. For instance, ChatGPT, which is based on a multi-layered deep neural network, has been important since it came out. As a large language model, it can be used for various natural language-processing tasks, and it has been widely applied in social media, customer service chatbots, and virtual assistants. Neural networks aim to solve complex problems, and they have been widely used in tackling optimization problems or mathematical programming. Optimization is a typical mathematical problem, and it is widely encountered in all engineering disciplines. Literally, it refers to finding an optimal/desirable solution. Optimization problems are complex and numerous, so approaches to solving these problems are always an active topic. Optimization approaches can be essentially distinguished into deterministic and stochastic. However, the former requires enormous computational resources. This is the motivation for exploring more efficient methods.

As a class of neural networks, recurrent neural networks (RNNs) have demonstrated great computational power, and they have been investigated in many engineering and scientific fields over the past decades. The authors of [3] proposed a gradient-based RNN (GBRNN) model for solving matrix inversion problems. However, there are some restrictions for methods under traditional neural networks, such as GBRNN. The ability of GBRNN to deal with time-varying problems is limited. In [4], the author verified that the residual error function of GBRNN will accumulate over time, which means it is unable to efficiently deal with time-varying problems. This paper focuses on a special RNN, named a zeroing neural network (ZNN), specifically for solving time-varying problems. Unlike the existing surveys, this paper presents a new classification method based on the performance index of ZNNs. According to the different performance abilities of ZNNs, it can be classified into the following three categories: (1) an accelerated-convergence ZNN, the type of ZNN that has fast convergence characteristics; (2) a noise-tolerance ZNN, the type of ZNN that has noise tolerance characteristics; (3) a discrete-time ZNN, the type of ZNN that can achieve higher computational accuracy and is more easily implemented in hardware. It is worth noting that these three types of ZNNs do not exist in isolation; they can be organically integrated.

As an optimization technique, swarm intelligence algorithms are driven by natural phenomena and behaviors, and they are particularly suited for problems that are non-linear, multi-modal, and have complex search spaces. These algorithms excel in exploring diverse regions of the solution space and efficiently finding near-optimal solutions. Similarly, many swarm intelligence algorithms have been applied in many scientific and engineering fields [5,6,7,8]. For example, a bio-inspired algorithm named the ant colony algorithm has been proposed for vehicle heterogeneity and backhaul mixed-load problems [9]. This algorithm is intended to jointly optimize the vehicle type, the vehicle number and travel routes, and minimize the total service cost. Through the introduction of intelligent algorithms, many fields have ushered in new developments and opportunities.

This paper aims to present a comprehensive survey of intelligence algorithms, including a neural network designed to solve time-varying problems, a swarm intelligence algorithm, and some practical fields that combine the rest of the intelligent algorithms. The algorithms’ origins, basic principles, recent advances, future challenges, and applications are described in detail. The paper is organized as follows. In Section 3, ZNNs are discussed in detail, including their origin, basic principle, operation mechanism, model variants, and applications. The rest of the intelligence algorithms are discussed in Section 4, including the genetic algorithm, the particle swarm algorithm, and some real applications. Conclusions are drawn in Section 5.

## 2. Related Work

There are review articles on neural networks, as well as bio-inspired algorithms, mostly devoted to the origins and inspirations of algorithms and categorizing existing algorithms. Ref. [10] classified bio-inspired algorithms into seven main groups: stochastic algorithms, evolutionary algorithms, physical algorithms, probabilistic algorithms, swarm intelligence algorithms, immune algorithms, and natural algorithms. In another example, some researchers have started from a specific domain and explored the study of algorithms applied to that domain. For example, ref. [11] started from the field of electrical drives and discussed the bio-inspired algorithms applied to this field. There have been numerous reviews of bio-inspired algorithms written in various ways. However, very few of them have focused on a specific algorithm. Therefore, starting with how to solve the optimization problem, this paper divides the optimization problem into a time-varying/dynamic optimization problem and a time-invariant/static optimization problem, highlighting a neural network ZNN for time-varying/dynamic optimization problems. For the rest of the algorithms, the authors prefer that the readers have some understanding of these algorithms and their origins, fundamentals, and applications.

ZNNs are a subfield of neural networks, and they belong to a neural network based on neurodynamics. To the best of our knowledge, only a few attempts have been made to comprehensively review ZNNs. In [12], the authors focused on mathematical programming problems in optimization problems by categorizing mathematical programming problems and discussing the ZNN models corresponding to different mathematical programming problems. Ref. [12] only explored the application of ZNN models to different mathematical programming problems and did not discuss neural networks such as ZNNs in depth. Ref. [13] exhaustively described the application areas involved in ZNNs, as well as the model variants, but like ref. [12], it did not provide a good introduction to ZNNs themselves, mention the origin of ZNNs, or mention the topology of ZNNs. The ZNN model is divided into different application areas. This classification model involves a lot of subjectivity and uncertainty. Compared to other works, this paper provides an in-depth overview of ZNNs, and it comprehensively introduces their origin, structure, operation mechanism, model variants, and applications. A novel classification of ZNNs is first proposed, categorizing models into accelerated convergence ZNNs, noise tolerance ZNNs, and discrete-time ZNNs, each offering unique advantages for solving time-varying optimization problems. The main contribution of this paper is that it identifies the structural topology of ZNNs, analyzes and explains the operating mechanism of ZNNs to some extent, and classifies ZNNs structurally.

**Remark 1.**  *Neural networks and bio-inspired optimization algorithms have a lot in common. Firstly, both neural networks and bio-inspired optimization algorithms are types of bio-heuristic algorithms; in essence, both types of algorithms are summarized from inspiration gained from nature. For example, Ref. [14] explored neural networks and bio-inspired optimization algorithms as two different classes of bio-heuristic algorithms and discussed their applications in the field of sustainable energy systems. Ref. [15] described a bio-inspired algorithm, i.e., the particle swarm algorithm, and neural networks in terms of concepts, methodology, and performance. Secondly, all the related previous research [9,16,17] has considered neural networks and bio-inspired optimization algorithms as the same concept, i.e., “computational intelligence”. Both neural networks and bio-inspired optimization algorithms are considered powerful computing tools.*

*Neural networks and bio-inspired optimization algorithms have many intersections and correlations. Firstly, bio-inspired optimization algorithms are widely used in neural network parameter optimization [18,19]. Especially in [20], the author paid special attention to the optimization of recurrent neural network parameters using bio-inspired optimization algorithms and provided a comprehensive analysis. Secondly, neural networks and bio-inspired optimization algorithms are used in common applications, and they are always used for comparison, such as comparing intelligent feature selection and classification [21,22], as well as structural health monitoring [23].*

*Recurrent neural networks are a class of neural networks, and we believe that it is feasible to combine them with bio-inspired optimization algorithms in one text. Particularly in this paper, from the perspective of solving optimization problems, the author classified optimization problems as dynamic/time-varying and static/time-invariant, and in particular, they introduced a recurrent neural network that has been widely used to solve dynamic/time-varying optimization problems.*

**Remark 2.**  *The writing logic of the paper revolves around how to solve the optimization problem. By observing the laws or habits of organisms in nature, researchers have been surprised to find that modeling the laws and habits of these organisms can be constructively helpful in solving practical problems. As a result, many such algorithms have been born, and they are collectively known as intelligent algorithms. Since these algorithms mainly realize computational functions, some researchers refer to them as “computational intelligence” [10,16,17]. In this paper, optimization problems are classified into time-varying/dynamic optimization problems and time-invariant/static optimization problems, and a class of neural networks for solving time-varying/dynamic optimization problems is described in detail in Section 2, while the rest of the bio-inspired algorithms used for optimization problems, including swarm intelligence algorithms and genetic algorithms, are described in Section 3.*

*In the field of application, neural networks and bio-inspired optimization algorithms have a broad and deep intersection. Bio-inspired optimization algorithms, especially swarm intelligence algorithms, are widely used for hyperparameter optimization and training process optimization for neural networks. In development trends, there is an increasing number of studies that have attempted to combine swarm intelligence algorithms with neural networks in order to create hybrid algorithms, for example, using PSO to optimize the architecture or parameters of convolutional neural networks [24]. Some research has focused on the co-evolution of swarm intelligence algorithms and neural networks, optimizing both simultaneously within the same system and promoting each to evolve towards better solutions. As future prospects, future research may develop more efficient swarm intelligence optimization methods, further enhancing the performance and training efficiency of neural networks. As technology advances, the combination of swarm intelligence algorithms and neural networks is expected to play a crucial role in more emerging fields, such as autonomous driving, smart manufacturing, and personalized medicine.*

**Remark 3.**  *Biomimetic algorithms, bio-inspired algorithms, and bio-inspired intelligence algorithms can be viewed as synonyms, all denoting algorithms that draw on the good design and functionality found in biological systems. Intelligent algorithms refer to a class of algorithms that use artificial intelligence techniques to solve complex problems. These algorithms often mimic intelligent behaviors observed in biological systems to find optimal or near-optimal solutions in complex environments. They possess adaptive, learning, and optimization capabilities that enable them to perform well in dynamic and uncertain situations. In addition to the bio-heuristic algorithms mentioned above, intelligent algorithms include several statistically or probabilistically inspired algorithms such as Bayesian optimization, random forest algorithms, and K-means algorithms. The conclusion is that bio-inspired algorithms are a subset of intelligent algorithms.*

## 3. Bio-Inspired Neural Network Models

### 3.1. Origin

The interior of each neuron cell is filled with a conducting ionic solution, and the cell is surrounded by a membrane of high resistivity. Ion-specific pumps maintain an electrical potential difference between the inside and the outside of the cell by transporting ions such as $K^{+}$ and ${\mathrm{Na}}^{+}$ across the membrane. Neuron cells respond to outside stimulation by dynamically changing the conductivity of specific ion species in synapses and cell membranes. Based on the response mechanisms of biological neuron cells, John Hopfield [25] used a neural dynamics equation to describe this change in conductivity. He applied his physical talents to the field of neurobiology in order to help people better understand how the brain works.

In 1982, Hopfield [26] introduced the concept of “computational energy”, and he proposed the Hopfield neural network (HNN) model. Two years later, he proposed the continuous-time HNN model [27], which pioneered a new way for neural networks to be used for associative memory and optimization calculations. This pioneering research work has a strong impetus to the study of neural networks. In 1985, Hopfield and Tank [28] proposed an HNN model for solving a difficult but well-defined optimization problem, the traveling-salesman problem, and used it to illustrate HNNs’ computational power. After that, numerous neural networks have been proposed, owing to this seminal work.

**Figure 1:**
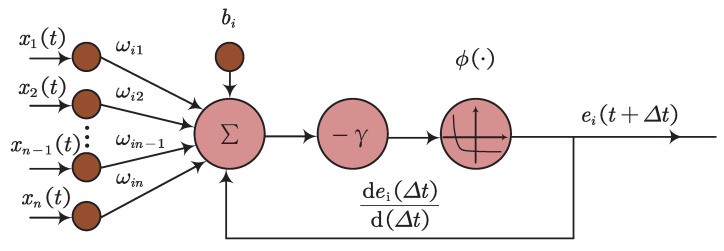
ZNN structure.

Similarly, Zhang introduced the concept of an “error function” and proposed a kind of RNN named a zeroing neural network (ZNN). ZNNs are easier to implement in hardware, and they belong to a non-training model. Essentially, the reason why ZNNs can effectively tackle time-varying problems is that they can utilize time-derivative information. For any given random initial input values, ZNNs operate in a neurodynamic manner, and they evolve in the direction of a decreasing error function, eventually reaching a steady state. The ZNN neural dynamics can be formulated as follows:

(1) $$\frac{dE(t)}{dt}=-\gamma \phi \left(E\left(t\right)\right),$$

where E(t) is the error function, γ>0 is the design parameter, and ϕ(·) denotes the activation function. The key step in designing the ZNN model is to construct an error function, E(t). For an arbitrary time-varying problem, WX(t)=b, the error function can be formulated as E(t)=WX(t)−b, where X(t) is the time-varying unknown variable to be solved. The structure of a ZNN is described in Figure 1, and input and output relationships can be described as follows:

(2) $$e_{i}\left(t+\Delta t\right)=e_{i}\left(t\right)+\frac{de_{i}\left(\Delta t\right)}{d\Delta t}\Delta t,$$

where $e_{i}=\sum_{j=1}^{n}\omega_{ij}x_{j}\left(t\right)+b_{i}$ is the *i*th neuron’s sub-error, $\omega_{ij}$ is the weight coefficient, and Δt refers to the next time. When all sub-error increments converge to zero, the system achieves stability, i.e., E(t)=WX(t)−b=0. Based on the superiority of ZNNs in real-time processing, many fruitful academic outcomes have been reported [29,30,31,32,33,34]. The following subsections discuss the convergence and stability of ZNNs, as well as model variants and applications.

### 3.2. Convergence and Stability

The key performance aspects of ZNNs are convergence and stability. Generally speaking, this can be proven via three approaches, i.e., Lyapunov theory, ordinary differential equations, and Laplace transformation.

**Proof.**  (**Based on Lyapunov theory**). For example, to solve a time-varying, nonlinear minimization problem, the target functions are f(x(t),t)∈R and $x\left(t\right)\in R^{n}$; ref. [35] designed an error function as

(3) $$E\left(t\right)=\frac{\partial f(x(t),t)}{\partial x(t)}.$$

Then, a Lyapunov function candidate is constructed:

(4) $$V\left(t\right)=\frac{1}{2}E^{T}\left(t\right)E\left(t\right),$$

it is obvious that V(t) is positive definiteness. Then, the formulation of its time derivative can be described as

(5) $$\dot{V}\left(t\right)=-\gamma E^{T}\left(t\right)E\left(t\right),$$

which is negative definiteness. That means the residual error of the ZNN model is globally converged to zero. Notably, by substituting the definition of the error function, E(t), the global convergence of all extant continuous-time ZNN models can be analyzed using a similar methodology. This analytical approach represents the predominant method for verifying continuous-time ZNN models, and it has been extensively investigated in [36,37,38,39]. □

**Proof.**  (**Based on an ordinary differential equation**). The ordinary differential equation is mainly used to verify the convergent speed of ZNN models activated via linear functions. For example, when considering the problem in [35], solving the *i*th subsystem of the design formula ${\dot{E}}_{i}=-\gamma E_{i}\left(t\right)$, it can lead to

(6) $$E_{i}\left(t\right)=E\left(0\right)\exp\left(-\gamma t\right),$$

where E(0) denotes the initial value of E(t). Finally, the residual error of the ZNN model globally and exponentially is set to zero. There has been extensive research presented in [40,41]. □

**Proof.**  (**Based on Laplace transformation**). Let us use Laplace transformation for $\dot{E}\left(t\right)=-\gamma E\left(t\right)$ and get

(7) sE(s)−E(0)=−γE(s),

and, further, have

(8) $$E\left(s\right)=\frac{E(0)}{s+\gamma }.$$

Due to γ>0, the final value theorem can be applied. Based on the final value theorem, we get

(9) $$\lim_{t\to \infty }E\left(t\right)=\lim_{s\to 0}sE\left(s\right)=\lim_{s\to 0}\frac{sE(0)}{s+\gamma }=0,$$

the proof is completed. This approach was studied in [42,43,44]. □

### 3.3. Accelerated Convergence

To solve some of the shortcomings of conventional neural networks, e.g., long training times and high computational resource consumption, an accelerated convergence neural network undoubtedly provides an effective solution to these issues. In past decades, there have been two main kinds of accelerated convergence neural networks: finite-time neural networks and predefined-time neural networks. What finite-time neural networks and predefined-time neural networks have in common is importing activation functions (AFs). For convenience, the main usages of AFs are listed as follows:

• General linear AF:
  (10) ϕ(x)=x.
• Power AF:
  (11) $$\phi \left(x\right)=x^{n},$$
  where *n* is an odd integer, and *n* > 3.
• Bipolar sigmoid AF:
  (12) $$\phi \left(x\right)=\frac{1-e^{-nx}}{1+e^{-nx}},$$
  where n>1.
• Hyperbolic sine AF:
  (13) $$\phi \left(x\right)=\frac{e^{nx}-e^{-nx}}{2}$$
  where n>1.
• Power-sigmoid AF:
  (14) $$\phi \left(x\right)=\left\{\begin{array}{cc} x^{n} & ,\;if\;\left|x\right|\leq 1\\ \frac{1+e^{-n}}{1-e^{-n}}\cdot \frac{1-e^{-nx}}{1+e^{-nk}} & ,\mathrm{ohterwise}. \end{array}\right.$$
• Constant-sign–bi-power AF:
  (15) $${\mathrm{sgn}}^{n}\left(x\right)=\left\{\begin{array}{cc} 1 & \mathrm{if}\;x>0;\\ 0 & \mathrm{if}\;x=0;\\ -1 & \mathrm{if}\;x<0. \end{array}\right.$$
• Sign–bi-power AF:
  (16) $$\mathrm{sbp}\left(x\right)={\mathrm{sgn}}^{n}\left(x\right)+{\mathrm{sgn}}^{1/n}\left(x\right),$$
  where sbp(·):R→R with the parameters n∈(0,1) and sgn^*n*^(·) defined as
  $${\mathrm{sgn}}^{n}\left(x\right)=\left\{\begin{array}{cc} |x^{n}| & \mathrm{if}\;x>0;\\ 0 & \mathrm{if}\;x=0;\\ -|x^{n}| & \mathrm{if}\;x<0. \end{array}\right.$$
• Weighted-sign–bi-power AF:
  (17) $$\mathrm{wsbp}\left(x\right):=\mu_{1}{\mathrm{sgn}}^{n}\left(x\right)+\mu_{2}{\mathrm{sgn}}^{1/n}\left(x\right)+\mu_{3}x,$$
  where $\mu_{1},\mu_{2},\mu_{3}$ are tunable positive design parameters.
• Nonlinear AF 1:
  (18) $$\phi \left(x\right)={\mathrm{sgn}}^{n}\left(x\right),$$
  where 0<n<1.
• Nonlinear AF 2:
  (19) $$\phi \left(x\right)=\xi {\mathrm{sgn}}^{n}\left(x\right)+\lambda x,$$
  where 0<n<1,ξ>0, and λ>0.

#### 3.3.1. Finite-Time Neural Network

The finite-time convergence of neural network models refers to the ability of a neural network to converge to a satisfactory state or achieve a predetermined level of performance within a finite period of time. In [45], Xiao proposed two nonlinear ZNN models based on nonlinear activation functions (Equations (18) and (19)) to efficiently solve linear matrix equations. According to Lyapunov theory, two such nonlinear ZNNs are proven to be convergent within finite time and the lower convergence bound. According to this paper, the convergence bound is

$$T_{u}\leq \max\left\{T_{u}^{-},T_{u}^{+}\right\}\leq \max\left\{\frac{|\eta^{-}{\left(0\right)}^{1-\xi }|}{\lambda (1-\xi )},\frac{|\eta^{+}{\left(0\right)}^{1-\xi }|}{\lambda (1-\xi )}\right\},$$

where $T_{u}$ denotes the convergence upper bound.

In [46], a ZNN with a specially constructed activation function was proposed and investigated to find the root of nonlinear equations. Then, comparing it with gradient neural networks, Xiao [47] pointed out that this model has better consistency in actual situations and a stronger ability in dynamical systems. The specially constructed activation function can be defined as Equation (16) and named the sign–bi-power function or Li function [48]. Due to its finite-time convergence, most researchers have employed it. For example, ref. [49] proposed an improved ZNN model for solving a time-varying linear equation system. Such a ZNN model is activated via an array of continuous sign–bi-power functions (Equation (15)). In [50], to solve dynamic matrix inversion problems, two finite-time ZNNs with the sign–bi-power activation function were proposed by designing two novel error functions. Furthermore, Lv [51] introduced a weighted sign–bi-power function (Equation (17)) to ZNNs. The ZNN model makes full use of all the items of the weighted sign–bi-power function, and thus, it obtains a lower upper bound for the convergence time.

Differing from the previous processing method, ref. [52] proposed a new design formula, which also can accelerate a neural network to finite-time convergence. This neural network achieves a breakthrough in convergence performance. According to this paper, an indefinite matrix-valued, time-varying error function, E(t), is defined as follows:

$$E\left(t\right)=A\left(t\right)X\left(t\right)-I\in R^{n\times n}.$$

Then, the new design formula for E(t) is proposed as follows:

$$\dot{E}\left(t\right)=-\gamma \varphi \left(\lambda_{1}E\left(t\right)+\lambda_{2}E^{q/p}\left(t\right)\right),$$

where φ(·) denotes an activation function array, and γ>0 is used to adjust the convergence rate with the design parameters $\lambda_{1}>0,\;\lambda_{2}>0$; *p* and *q* denote positive odd integers and satisfy p>q.

#### 3.3.2. Predefined-Time Neural Network

The predefined-time convergence of a neural network refers to the ability of a neural network to converge to a satisfactory state or achieve a predetermined level of performance within a predefined time. In some actual applications [53,54] that require the fulfillment of strict time constraints, there is a requirement that a neural network model can guarantee a timely convergence. For instance, the common problem in numerous fields of science and engineering is solving the dynamic-matrix square root (DMSR) [55,56]. It is preferable to solve the DMSR via a neural network with explicitly and antecedently definable convergence time. Li [57] proposed a predefined-time neural network, the convergence time of which can be explicitly defined as a prior constant parameter. In this paper, the maximum predefined time can be formulated as

(20) $$T_{\max}=\frac{1}{\lambda (1-a)}+\frac{1}{\mu (b-1)},$$

where λ>0,μ>0,0<a<1 and b>1 are prior constant parameters. Similarly, Xiao [58] presented and introduced a new ZNN model using a versatile activation function for solving time-dependent matrix inversion. Unlike the existing ZNN models, the proposed ZNN model not only converges to zero within a predefined, finite time but also tolerates several noises in solving the time-dependent matrix inversion. Differing from the method of using AFs, ref. [59] proposed a very parameter-focused neural network for improving the ZNN model’s convergence speed, which is more compatible with the characteristics of the actual hardware parameter.

### 3.4. Noise Tolerance Neural Network

Noise in a neural network refers to the uncertainty or randomness in data, which can originate from various sources, including sensor errors, environmental interference, and incidental factors during data collection. In neural networks, noise can have significant impacts on model training and performance. Jim [60] first discussed the impact of noise on recurrent neural networks and pointed out that the introduction of noise will also help improve the convergence and generalization performance of recurrent neural network models. However, for the most part, the impact of noise on neural networks is negative. To eliminate the impact of noise, most researchers are devoted to designing a noise filter, such as a Gaussian filter [61]. This has achieved major success in image-recognition denoising [62]. But, in practical engineering applications, it is hard to gauge in advance which noise it is and, thus, hard to design the corresponding filter. Then, researchers focused on improving the robustness of neural networks. Liao [63] analyzed the traditional ZNN model and verified that it has a tolerance for low-amplitude noise during multiple noise interferences. There are two main approaches to tolerating noise interference: (1) introducing AFs and (2) introducing integral terms. In [64,65], some AFs were introduced to improve the robustness of neural networks. To tolerate harmonic noise interference, a Li activation function [66] was introduced in order to further improve the convergence rate and robustness. The convergence and robustness of harmonic noises of the proposed neural network models were proven through theoretical analyses.

Research [67] first proposed a noise-tolerance ZNN model with a single integral term for solving the time-varying matrix inversion problem, which can be mathematically formulated as follows:

(21) $$\dot{E}\left(t\right)=-\gamma E\left(t\right)-\mu \int_{0}^{t}E\left(\tau \right)\;d\tau .$$

where γ and μ∈R>0. Essentially, a single-integral-structure ZNN model uses the error integration information to mitigate constant bias errors. No matter how large the matrix-form constant noise is, these kinds of ZNN models can converge to the theoretical solution with an arbitrarily small residual error. These design models can serve as a fundamental framework for future research on solving time-varying problems, and they can open a door to solving time-varying problems with constant noise. Based on [67], the research [68] presented a ZNN model with a single term to compute the matrices’ inversion, which was subjected to null space and specific range constraints under various noises. This is an important topic to further enhance the robustness of ZNN models. Thus, Liao [29] further extended the single integral framework in [67] and designed a double-integral-structure ZNN model to solve various types of time-varying problems, whose structure can be described as follows:

(22) $$\dot{E}\left(t\right)=-3\gamma E\left(t\right)-3\gamma^{2}\int_{0}^{t}E\left(\tau \right)\;d\tau -\gamma^{3}\int_{0}^{t}\int_{0}^{\tau }E\left(\iota \right)\;d\iota d\tau .$$

Then, in [69], to solve matrix inversion problems with linear noise rejection, this paper illustrated that a double-integral-structure ZNN model can effectively tolerate linear noise. Similarly, to enhance robustness in solving matrix inversion problems, ref. [70] proposed a variable-parameter, noise-tolerant ZNN model. Compared with a single-integral-structure ZNN model for matrix inversion, numerical simulations revealed, a double-integral-structure ZNN model has better robustness under the same external noise interference.

### 3.5. Convergence Accuracy

As mentioned before, a series of continuous-time ZNN models have been proposed and designed to solve different problems. However, the fact is that step sizes in simulating continuous-time systems are variable, while a digital computer often requires constant time steps. It is hard to implement continuous-time ZNN models using digital computers. Additionally, continuous-time ZNN models always work in ideal conditions, i.e., it is supposed that neurons communicate and respond without any delay. Nevertheless, because of the existence of the sampling gap, a time delay is inevitable; it unavoidably leads to a reduction in the computing accuracy of ZNN models. Thus, researchers proposed and investigated discrete-time ZNN models. For example, Xiang [71] proposed a discrete-time ZNN model for solving dynamic matrix pseudoinversion. There are some challenges in constructing discrete-time ZNN model, and they are listed as follows.

• In view of time, any time-varying problem can be considered a causal system. The computation should be based on existing data, which include present and/or previous data. For instance, when solving the time-varying matrix inversion problem discretely, at time instant $t_{k}$, we can only use known information, such as $A(t_{k})$ and $\dot{A}\left(t_{k}\right)$, not unknown information, such as $A(t_{k+1})$ and $\dot{A}\left(t_{k+1}\right)$, to compute the inverse of $A(t_{k+1})$, i.e., $X(t_{k+1})$. Therefore, a fundamental requirement for constructing the discrete-time ZNN model is that unknown data cannot be used.
• This means that a potential numerical differentiation formula for discretizing a continuous-time ZNN model should have and should only have one point ahead of the target point. Thus, in the process of discretizing the continuous-time ZNN model using the numerical differentiation formulation, the discrete-time ZNN model has only one unknown point, $X(t_{k+1})$, to be computed. Thus, no matter how minimal the truncation error of each formula is, discrete-time ZNN models cannot be constructed using backward and multiple-point central differentiation rules. Only the one-step-ahead forward differentiation formulas can be considered for discrete-time ZNNs.
• Time is valuable for solving time-varying problems. It is crucial to design a very straightforward discrete-time ZNN model with minimal time consumption.

In [72], Zhang proposed the first discrete-time ZNN model for solving constant matrix inversion. This model built a critical bridge between discrete-time ZNN frameworks and traditional Newton iteration. At the beginning, ref. [73,74] constructed the discrete-time ZNN models using the Euler forward difference. Notably, the error pattern of discrete-time ZNN models derived from the Euler forward difference approach is $O(\tau^{2})$, where τ is the sampling interval. For instance, the Euler-type discrete-time ZNN model for time-varying matrix inversion is directly formulated as [75]

(23) $$X_{k+1}=X_{k}-\tau X_{k}{\dot{A}}_{k}X_{k}-hX_{k}\left(A_{k}X_{k}-I\right),$$

where *k* denotes the iteration index, and h=τγ>0. Especially by omitting the term $\tau X_{k}{\dot{A}}_{k}X_{k}$ and setting h=1, the Euler-type discrete-time ZNN model (24) is simplified to

(24) $$X_{k+1}=X_{k}-X_{k}\left(A_{k}X_{k}-I\right),$$

which aligns with the traditional Newton iteration. Hence, the traditional Newton iteration can be considered a special case of the Euler-type discrete-time ZNN model (24). Furthermore, a linkage between the Getz–Marsden dynamic system and discrete-time ZNN models was established in [76]. To enhance the accuracy of ZNN model discretization, a Taylor-type numerical differentiation formula was proposed in [77] for first-order derivative approximation. This formula exhibits a truncation error of $O(\tau^{2})$, and it is expressed as:

$$f^{\prime }\left(t_{k}\right)=\frac{2f\left(t_{k+1}\right)-3f\left(t_{k}\right)+2f\left(t_{k-1}\right)-f\left(t_{k-2}\right)}{2\tau }+O\left(\tau^{2}\right),$$

subsequently, a novel, Taylor-type, discrete-time ZNN model was developed for time-varying matrix inversion,

$$X_{k+1}=-\tau X_{k}{\dot{A}}_{k}X_{k}-hX_{k}\left(A_{k}X_{k}-I\right)+\frac{3}{2}X_{k}-X_{k-1}+\frac{1}{2}X_{k-2}.$$

This Taylor-type, discrete-time ZNN model converges to the theoretical solution of the time-varying problem with a residual error of $O(\tau^{3})$

Recently, a numerical differentiation rule with a truncation error of $O(\tau^{3})$ was established in [78] for first-order derivative approximation. By utilizing this new formula, a five-step discrete-time ZNN model was proposed for time-varying matrix inversion, with a residual error of $O(\tau^{4})$. It is imperative to note that discrete-time ZNN models can be considered time-delay systems, and consequently, a high value of *h* may induce oscillations in the model. To mitigate instability, reducing the value of *h* is a direct remedy. However, decreasing *h* significantly decelerates the convergence of discrete-time ZNN models.

### 3.6. Time-Varying Linear System Solving

Time-varying linear systems have extensive applications in engineering fields such as circuit design [79], communication systems [80,81,82], and signal processing [83,84]. Many engineering problems can be effectively modeled as time-varying linear systems [85,86]. Time-varying linear systems can be constructed using a series of time-varying matrices that store system information, which changes over time.

One notable contribution in this field is the work of Lu [87], who proposed a novel ZNN model for solving time-varying underdetermined linear systems. This model satisfied variable constraints and effectively converged the residual errors. Through extensive theoretical analyses and numerical simulations, the author demonstrated the effectiveness and validity of the proposed ZNN model and showcased its applicability in controlling the PUMA560 robot under physical constraints. Additionally, Xiao [88] designed a ZNN model specifically for time-varying linear matrix equations. Their study included a theoretical analysis of the maximum convergence time, and it concluded with exceptional performance in solving time-varying linear equations. Zhang [89] proposed a varying-gain ZNN model for solving linear systems, which can be represented as A(t)B(t)C(t)=D(t). Due to the varying properties parameter, this ZNN model can achieve finite-time convergence. Moreover, several other ZNN models, proposed in [90,91], have been presented for solving time-varying Sylvester equations of the form A(t)B(t)−B(t)A(t)=−C(t).

Similarly, a complex time-varying linear system has also been mainly discussed and investigated. The complex number domain is widely and profoundly used in science and engineering, which not only exists as a mathematical tool but also plays a key role in solving many practical problems. For the online solving of complex time-varying linear equations, ref. [92] proposed and investigated a neural network. This proposed neural network adequately utilizes time-derivative information for time-varying complex matrix coefficients, and it was theoretically proven that it can converge to the theoretical solution with finite time. In [93], a complex-valued, nonlinear, recurrent neural network was designed for time-varying matrix inversion solving in complex number fields. This paper proposed a complex-valued, nonlinear ZNN model that was established on the basis of a nonlinear evolution formula and that possessed better finite-time convergence. Long [94] mainly discussed the finite-time stabilization of a complex-valued ZNN. This article investigated the finite-time stabilization of a complex-valued ZNN with a proportional delay via the direct analysis method without separating the real and imaginary parts of complex values. To achieve higher precision and a higher convergence rate, ref. [95] proposed a new ZNN model. Compared with the GBRNN model and the existing ZNN models, the illustrative results showed that the new ZNN model has higher precision and a higher convergence rate.

### 3.7. Time-Varying Nonlinear System Solving

Nonlinear systems are prevalent in many real-world applications, and real-time solutions for nonlinear systems have been a hot topic. In [42], a continuous-time ZNN model was presented and investigated for online time-varying nonlinear optimization. This paper focused on the convergence of continuous-time ZNN models, and it theoretically verified that the continuous-time ZNN model is provided with a global exponential convergence property. For the purpose of satisfying both finite-time convergence and noise tolerance, Xiao [96] proposed a finite-time robust ZNN to solve time-varying nonlinear minimization under various external disturbances. Ref. [97] proposed a finite-time, varying-parameter, convergent-differential ZNN for solving nonlinear optimization. The proposed ZNN model has super exponential convergence, finite-time convergence, and strong robustness.

Quadratic minimization is a fundamental problem in nonlinear optimization, and it has wide applicability. The goal of quadratic minimization is to find values of variables that minimize a quadratic objective function subject to certain constraints. Ref. [98] proposed a segmented variable-parameter ZNN model for solving time-varying quadratic problems. They achieved strong robustness by keeping the time-varying parameters stable. The general time-varying quadratic problem can be expressed as follows:

(25) $$\begin{array}{cc} \min.\; & x^{T}\left(t\right)H\left(t\right)x\left(t\right)-f^{T}\left(t\right)x\left(t\right),\\ s.t.\; & A(t)x(t)=\mu (t),\\ \; & B(t)x(t)⩽\upsilon (t), \end{array}$$

where the vector $x\left(t\right)\in R^{n}$ is the solution that should be tackled using the model, $A\left(t\right)\in R^{m\times n}$, $B\left(t\right)\in R^{w\times n}$, $f\left(t\right)\in R^{n}$, $\mu \left(t\right)\in R^{m}$, $H\left(t\right)\in R^{n\times n}$ is a symmetric positive definite matrix, and $\upsilon \left(t\right)\in R^{n}$; these time-varying coefficients are known. In [99], a single integral structure with a nonlinearly activated function ZNN model was proposed to solve dynamic quadratic minimization with additive noises considered. Differing from the above-mentioned ZNN model, ref. [100] proposed a new design formula. Based on this formula, a finite-time and noise-tolerance ZNN was proposed for solving time-varying quadratic optimization problems under various levels of additive noise interference.

### 3.8. Robot Control

As an effective approach to solving time-varying problems, ZNNs have been widely applied to robot control, such as redundant manipulators [101] and multiple-robot control [102,103]. For example, because the end-effector task may be incomplete due to a manipulator’s physical limitations or space limitations, it is important to adjust the manipulator configuration from one state to another state. In [104], Tang proposed a refined self-motion control scheme based on ZNNs, which can be described as follows:

$$\begin{array}{cc} \mathrm{minimize}\; & {∥\Omega \left(t\right)+q\left(t\right)∥}_{2}^{2}/2,\\ subject\;to\; & J\left(\Omega \left(t\right)\right)\dot{\Omega }\left(t\right)=-\gamma_{1}\left(F\left(\Omega \left(t\right)\right)-F\left(\Omega \left(0\right)\right)\right),\\ \; & A^{-}\leq \dot{\Omega }\leq A^{+},\\ \mathrm{with}\; & q\left(t\right)=\gamma_{2}t\left(\Omega \left(t\right)-\Omega_{g}\right),\\ \; & A^{-}\left(t\right)=\max\left\{\omega^{-}\left(t\right)+k\left(\omega^{-}\left(t\right)-\Omega \left(t\right)\right),\theta^{-}\left(t\right)\right\},\\ \; & A^{+}\left(t\right)=\max\left\{\omega^{+}\left(t\right)+k\left(\omega^{+}\left(t\right)-\Omega \left(t\right)\right),\theta^{+}\left(t\right)\right\}, \end{array}$$

where $\dot{\Omega }\left(t\right)\in R^{n}$ and $\Omega \left(t\right)\in R^{n}$ are the joint-angle-velocity vector and joint-angle vector, respectively; $\Omega_{g}$ denotes the given joint-angle vector; Ω(0) denotes the initial joint-angel vector; ${∥\cdot ∥}_{2}$ is the 2-norm of the vector; $q\left(t\right)\in R^{n}$ is defined according to the self-motion task; and J(Ω)=∂F(Ω)/∂Ω is the Jacobian matrix. $\gamma_{1},\;\gamma_{2}$ are the positive design parameters, and *k* is used to scale the magnitude of the manipulators. In addition, $\theta^{-}$ and $\theta^{+}$ denote the time-varying joint-angle lower bound and upper bound; $\omega^{-}$ and $\omega^{+}$ represent the joint-angle-velocity lower bound and upper bound. This self-motion can adjust the manipulator configuration from the initial state to the final state and keep the end effector immobile at its current orientation or position. Considering the higher accuracy, ref. [105] proposed a discrete-time ZNN model to solve the motion planning of redundant manipulators.

To effectively manage and control muti-robots, Liao [106] proposed a strategy for real-time control. This strategy achieves the real-time measuring and minimizing of inter-robot distances, which can be formulated as

(26) $$\min\sum_{j=1}^{N}\left((\right|P_{j}\left(t\right)-P_{j+1}{\left(t\right)|}^{2}+|P_{j}\left(t\right)-P_{j-1}{\left(t\right)|}^{2})/2),$$

where the complex number $P_{j}=x_{j}+y_{j}i$ is the position information of the *j*th follower robot, the real part $x_{j}$ and the imaginary part $y_{j}$ represent the *x*-coordinate and *y*-coordinate, and $P_{j-1}\left(t\right),\;P_{j+1}$ are two neighboring robots. Based on leader–follower frameworks, the desired formation is merely known by leader robots, and follower robots just follow the motion of the leaders (this paper chose the robots at the edges as leaders). Furthermore, to verify the correctness of this strategy, the author employed a wheeled mobile robot, and the kinematic model can be described as

$$\left\{\begin{array}{c} \dot{x}\left(t\right)=\left(\nu_{r}\left(t\right)+\nu_{l}\left(t\right)\right)\cos\omega \left(t\right)/2\\ \dot{y}\left(t\right)=\left(\nu_{r}\left(t\right)+\nu_{l}\left(t\right)\right)\sin\omega \left(t\right)/2\\ \dot{\omega }\left(t\right)=\left(\nu_{r}\left(t\right)-\nu_{l}\left(t\right)\right)/2. \end{array}\right.$$

Then, according to *differential flatness*, the left- and right-wheel velocity can be represented with finite-order differentials of the position information, which can be formulated as follows:

$$\left\{\begin{array}{c} \nu_{r}\left(t\right)=\sqrt{{\dot{x}}^{2}\left(t\right)+\dot{y}\left(t\right)}+\frac{\ddot{y}\left(t\right)\dot{x}\left(t\right)-\dot{y}\left(t\right)\ddot{x}\left(t\right)}{{\dot{x}}^{2}\left(t\right)+{\dot{y}}^{2}\left(t\right)}\\ \nu_{l}\left(t\right)=\sqrt{{\dot{x}}^{2}\left(t\right)+\dot{y}\left(t\right)}-\frac{\ddot{y}\left(t\right)\dot{x}\left(t\right)-\dot{y}\left(t\right)\ddot{x}\left(t\right)}{{\dot{x}}^{2}\left(t\right)+{\dot{y}}^{2}\left(t\right)}. \end{array}\right.$$

To achieve real-time control, the author constructed a complex ZNN model to find the real-time positions. Compared to the GBRNN model, this model can eliminate a large lagging error.

### 3.9. Discussion and Challenges

Overall, research on ZNNs has achieved considerable success in solving time-varying problems. However, there are still many challenges to be solved in the future. This paper provides some prospective suggestions for future directions.

(1) Keep exploring and discovering more useful, one-step-ahead numerical differential formulas to construct discrete-time ZNNs. One of the goals is that further discrete-time ZNN models possess larger step sizes and higher computational accuracy. Larger step sizes consume less computation time. Such advancements align closely with the development of applied mathematics and computational mathematics.
(2) How to achieve faster convergence still remains an open problem. Additionally, it is meaningful in the development of ZNNs to understand how to achieve convergence conditions.
(3) Apart from global stability, more theory is also needed to improve robustness.

All of these future developments will coincide with the advancement of mathematical theory, particularly in applied mathematics and computational mathematics. For instance, it is necessary to derive new models for addressing inequality constraints in time-varying optimization problems. These advancements will parallel the progress in computational mathematics, which is crucial for constructing and developing neural networks. It is important to recognize that different types of neural networks, such as discrete-time ZNN models or accelerated-convergence ZNN models, each have their own applicable ranges. Therefore, it is unrealistic to expect that an isolated ZNN model can solve all computational problems.

## 4. Intelligence Algorithms with Applications

Bio-inspired intelligence algorithms can be categorized into two specific categories, genetic algorithms and swarm intelligence algorithms. This section mainly discusses one of the classic evaluation algorithms, i.e., genetic algorithms, and one of the classic swarm intelligence algorithms, i.e., the particle swarm optimization algorithm. And it introduces their origins, design process, and basic principles. It also introduces a number of swarm intelligence algorithms, such as the ant colony optimization algorithm, an artificial fish swarm algorithm, and the Harris hawks optimizer. Then, we select some practical domains, i.e., gene feature extraction, intelligence communication, and the image process, to emphasize the applicability of intelligent algorithms.

### 4.1. Bio-Inspired Intelligence Algorithm

Bio-inspired optimization algorithms draw inspiration from biological evolution and swarm intelligence, aiming to solve complex optimization problems. These algorithms leverage the principles underlying biological systems, such as evolution, swarm intelligence, and natural selection, to tackle optimization, search, and decision-making tasks across diverse domains.

One prominent category of bio-inspired algorithms is evolutionary algorithms, which include genetic algorithms, evolutionary strategies, evolutionary programming, and genetic programming. These algorithms simulate the process of natural evolution, where candidate solutions/individuals evolve and adapt over successive generations through mechanisms such as selection, crossover/reproduction, and mutation, ultimately converging toward optimal or near-optimal solutions.

Another is swarm intelligence algorithms, which are inspired by the collective behavior of decentralized, self-organized systems observed in nature, such as ant colonies and bird flocks [107,108]. Swarm intelligence algorithms include particle swarm optimization, the egret swarm optimization algorithm [109], the beetle antennae search algorithm [110,111], and the gray wolf algorithm [112], which mimic the cooperative behaviors of social insects to efficiently explore solution spaces and find high-quality solutions.

These intelligence algorithms offer the following advantages:

• **Distributed robustness:** Individuals are distributed across the search space, and they interact with each other. Due to the lack of a centralized control center, these algorithms exhibit strong robustness. Even if certain individuals fail or become inactive, the overall optimization process is not significantly affected.
• **Simple structure and easy implementation:** Individuals have simple structures and behavior rules. They typically only perceive local information and interact with others through simple mechanisms. This simplicity makes the algorithms easy to implement and understand.
• **Self-organization:** Individuals exhibit complex self-organizing behaviors through interactions and information exchanges. The intelligent behavior of the entire swarm emerges from the interactions among simple individuals.

#### 4.1.1. Genetic Algorithm

**Figure 2:**
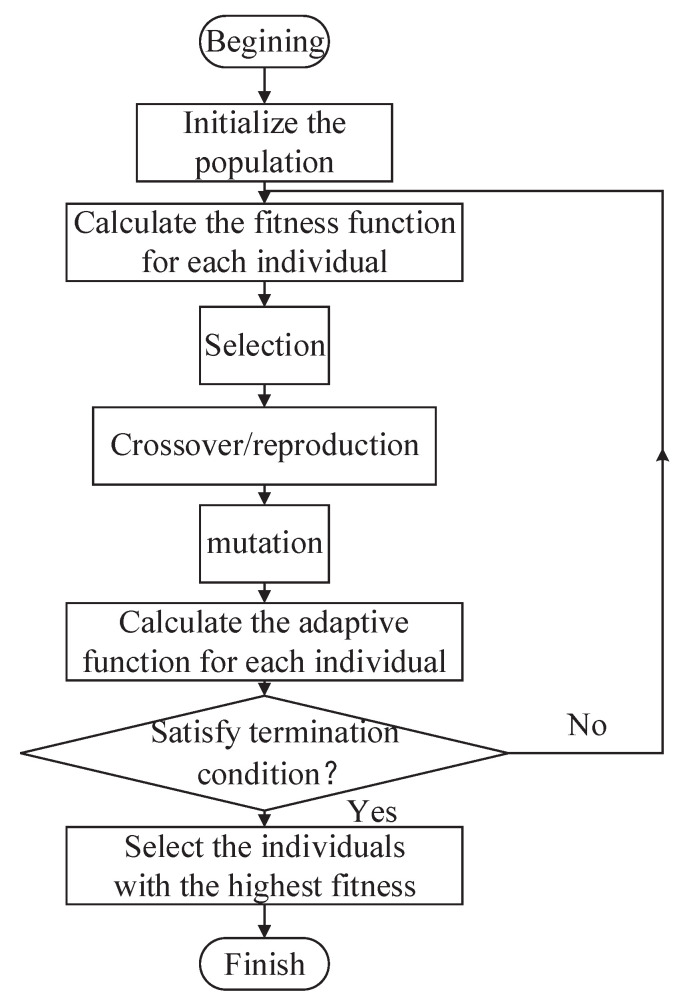
General procedure of GA.

In 1975, Holland [113] first proposed the genetic algorithm (GA), which was inspired by natural selection. As the most successful type of evolutionary algorithm, GA is a stochastic optimization algorithm with a global search potential. Essentially, GA emulates the process of natural selection in finding optimal solutions for complex optimization and search problems. The core idea of GA is the concept of the survival of the fittest. The fitness function is used to measure the merits of individuals, and the individuals with better performance have a higher probability of being selected to produce offspring in the next generation. Through the iterative process of reproduction and selection, GA gradually improves the solution set and converges toward the optimal or near-optimal solution. The design procedure can be described as follows, and the flow diagram is shown in Figure 2.

(1) Initialize the population.
(2) Calculate the fitness function for each individual.
(3) Select individuals with a high fitness function, and let them undergo crossover/reproduction and mutation to generate offspring.
(4) Calculate the fitness function for each individual.
(5) If the termination condition is satisfied, select the individual with the highest fitness, or else return to step (2).

Due to its remarkable performance in optimization, GA has been applied in many optimization problems, especially where the search space is large, complex, or poorly known. Ou [114] developed a new hybrid knowledge extraction framework by combining GA and backpropagation neural networks. The efficacy of the framework was demonstrated through a case study using the Wisconsin breast cancer dataset. Furthermore, Li [115] investigated the application of the harmonic search algorithm in this framework.

#### 4.1.2. Particle Swarm Optimization Algorithm

**Figure 3:**
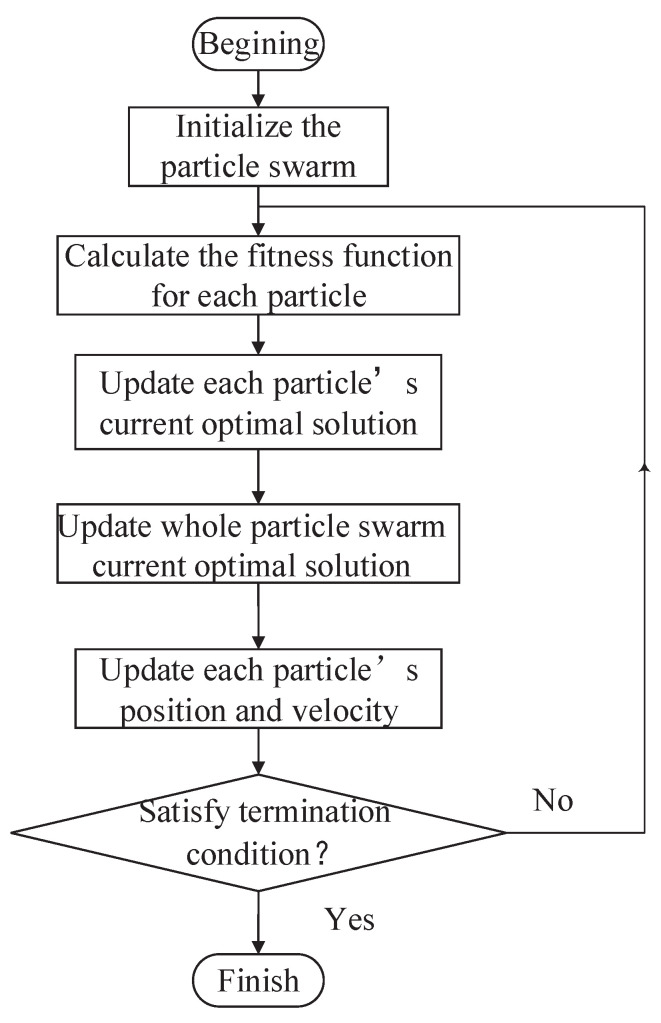
General procedure of PSO.

In 1995, by observing the social behavior of birds flocking and searching for food, Kennedy and Eberhart proposed particle swarm optimization (PSO) algorithm, which is stochastic and computational-intelligence-oriented [116]. ”Particles” usually refers to population members with an arbitrarily small mass and volume. Each particle denotes a solution with four vectors, including the current position, the current optimal solution, the current optimal solution found via its neighbor particles, and velocity. In each iteration, each particle updates its current position in the search space based on the current optimal position and the current neighbor particle’s optimal position. The renewal principle of each particle’s position and velocity can be described as follows:

(27) $$\begin{array}{cc} \; & P_{j+1}^{i}=P_{j}^{i}+\nu_{j+1}^{i}\\ \; & \nu_{j+1}^{i}=\nu_{j}^{i}+a_{1}b_{1}\left(P^{i*}-P_{j}^{i}\right)+a_{2}b_{2}\left(H^{*}-P_{j}^{i}\right) \end{array}$$

where $P_{j+1}^{i}$ denotes *i*th particle’s position in *j*th iteration; $\nu_{j}^{i}$ denotes the *i*th particle’s velocity in the *j*th iteration; $P^{i*}$ denotes *i*th particle’s current optimal position; $H^{*}$ denotes whole particles’ optimal positions; $a_{1}\;a_{2}$ denotes cognitive and social parameters; and $b_{1}\;b_{2}\in \left[0\;1\right]$. The design procedure can be described as follows, and the flow diagram is shown in Figure 3.

(1) Initialize the particle swarm.
(2) Calculate the fitness function for each particle.
(3) Update each particle’s current optimal solution.
(4) Update the whole particle’s current optimal solution.
(5) Update each particle’s position and velocity.
(6) If the termination condition is satisfied, select the individual with the highest fitness or else return to step (2).

Due to the PSO algorithm’s simple concept, easy implementation, computational efficiency, and unique searching mechanism, it has extensive applications in various engineering optimizations. To solve the problem associated with Takagi–Sugeno fuzzy neural networks, Peng [117] proposed an enhanced chaotic quantum-inspired PSO algorithm. This algorithm effectively solves problems such as a slow convergence time and a long calculation time. Additionally, Yang [118] proposed an improved PSO (IPSO) algorithm to identify parameters of the Preisach model for modeling hysteresis phenomena. Compared with traditional PSO methods, IPSO is provided with superior convergence, less computation time, and higher accuracy.

### 4.2. Ant Colony Optimization Algorithm

The ant colony optimization (ACO) algorithm was first introduced by M. Dorigo and colleagues in 1996 as a nature-inspired, meta-heuristic method aimed at solving combinatorial optimization problems. This algorithm draws inspiration from stigmergy, where communication within a swarm is achieved through environmental manipulation. It has been demonstrated that ants communicate by depositing pheromones on the ground or objects to send specific signals to other ants. Various pheromones are used for different tasks within a colony. For example, ants mark distinct paths from their nests to a food source to guide other ants for transportation. The shortest path is naturally selected because longer paths allow more time for pheromone evaporation before re-deposition.

In the ACO’s original version, the problem must be represented as a graph, where each ant represents a tour, and the goal is to identify the shortest one. There are two key matrices: distance and pheromones, which ants use to update their routes. Through multiple iterations and updates of these matrices, a tour is established that all ants follow, which is considered the best solution for the problem. ACO has various adaptations that can address problems with continuous variables, constraints, multiple objectives, and more. Bullnheimer [119] introduced an innovative rank-based variant of the ant system. In this version, the paths of the ants are sorted from the shortest to the longest after each iteration. The algorithm assigns different weights based on the path length, with shorter paths receiving higher weights. Hu [120] developed a new ACO called the “continuous orthogonal ACO” to solve continuous problems, using a pheromone deposition mechanism that allows ants to efficiently search for solutions. Gupta [121] introduced the concept of depth into a recursive ACO, where depth determines the number of recursions, each based on a standard ant colony algorithm. Gao [122] combined the K-means clustering algorithm with the ACO and introduced three immigrant schemes to solve the dynamic location routing problem. Hemmatian [123] applied an elitist ACO algorithm to the multi-objective optimization of hybrid laminates, aiming to minimize the cost during the computation process.

### 4.3. Artificial Fish Swarm

In nature, fish can identify more nutritious areas by either searching individually or following others, and areas with more fish tend to be richer in nutrients. In 2002, Li [124] initially introduced a stochastic, population-based AFS optimization algorithm. The algorithm’s core idea is to imitate fish behaviors like swarming, preying, and following, combining these with a local search to generate a global optimum [125]. The AFS algorithm generally has the same advantages as genetic algorithms, while the AFS algorithm can reach faster convergence and needs fewer parameter adjustments. Unlike genetic algorithms, AFS does not involve crossover and mutation processes, making it simpler to execute. It operates as a population-based optimizer: the system begins with a set of randomly generated potential solutions and iteratively searches for the best one [126]. Due to random search and the parallel optimization method, AFS has global search capabilities and fast convergence. The fish group is represented by a set of points or solutions, with each agent symbolizing a candidate solution. The feasible solution space represents the “waters” where artificial fish move and search for the optimum. The algorithm reaches the optimal solution through survival, competition, and coordination mechanisms. In the AFS algorithm, the swarm behavior is characterized by movement towards the central point of the visual scope. Numerous researchers have developed and improved the AFS algorithm, resulting in a variety of novel variants. For example, a binary AFS was proposed to solve 0–1 multidimensional knapsack problems. In this approach, a 0/1-bit binary string represents a point, and each bit of a trial point is generated by copying the relevant bit from itself or another specified point with equal probability. Zhang introduced a Pareto-improved AFS algorithm for addressing a multi-objective fuzzy disassembly-line-balancing problem, which helped reduce uncertainty. To enhance the global search ability and convergence speed, Zhu proposed a new quantum AFS algorithm based on principles of quantum computing, such as quantum bits and quantum gates.

### 4.4. Harris Hawks Optimizer

The Harris hawks optimizer (HHO) was developed by Heidari [127], and it is based on the natural hunting strategies of Harris hawks. This algorithm uses different equations to update the positions of hawks in a search space, emulating the different hunting techniques these birds use to capture prey. The following equation is used to provide exploratory behavior for HHO [127]:

$$X\left(t+1\right)=\left\{\begin{array}{cc} X_{s}\left(t\right)-a_{1}\left|X_{s}\left(t\right)-2a_{2}X\left(t\right)\right| & \mathrm{for}\;b\geq 0.5\\ \left(X_{p}\left(t\right)-X_{mp}\left(t\right)\right)-a_{3}\left(L+a_{4}\left(U-L\right)\right) & \mathrm{for}\;b\leq 0.5, \end{array}\right.$$

where X(t) denotes the position in the *t*th iteration, $X_{p}\left(t\right)$ is the prey position, $a_{1},\;a_{2},\;a_{3},\;a_{4}$ and *b* are a random number in [0,1], *L* and *U* are the parameters’ lower and upper bounds, respectively, $X_{s}\left(t\right)$ indicates a hawk randomly selected from the current population, and $X_{mp}\left(t\right)=\frac{1}{N}\sum_{i=1}^{N}X_{i}\left(t\right)$ represents the mean position of the current population of hawks. The exploitation algorithm employs two distinct besieging strategies: soft and hard:

(28) $$\begin{array}{cc} \; & X\left(t+1\right)=\bar{X}\left(t\right)-a_{5}\left|CX_{p}\left(t\right)-X\left(t\right)\right|,\\ \; & \bar{X}\left(t\right)=X_{p}\left(t\right)-X\left(t\right),\\ \; & X\left(t+1\right)=X_{p}\left(t\right)-a_{5}\left|\bar{X}\left(t\right)\right|, \end{array}$$

where $\bar{X}\left(t\right)$ represents the distance between the solution and the prey at the *t*th iteration, $a_{5}$ and *C* are random variables.

### 4.5. The Rest of the Swarm Intelligence Algorithms

Swarm intelligence algorithms are predominantly derived from simulations of natural ecosystems, especially those based on insects and animals. These algorithms are optimized by simulating the processes of foraging or information exchange within these biomes, and most utilize directional iterative methods based on probabilistic search strategies. With the progression of swarm intelligence, optimization algorithms have transcended merely incorporating biological population traits. Some studies have introduced human biological characteristics into swarm intelligence algorithms, such as the human immune system [128]. Recent innovations in swarm intelligence algorithms, as well as enhancements to classical algorithms, mainly aim at reducing parameters, streamlining processes, accelerating computation speeds, and improving search capabilities, particularly for high-dimensional and multi-objective optimization problems [129]. The application domains of swarm intelligence algorithms are expanding, with their usage guiding further development directions.

In addition to the well-known swarm intelligence algorithms, many extended algorithms have also been extensively discussed and utilized. The “cuckoo search” algorithm, inspired by the brood parasitic behavior of some cuckoo species and the Lévy flight behavior of birds and fruit flies, addresses optimization problems [130]. Pigeon-inspired optimization, used for air–robot path planning, employs a map and compass operator model based on the magnetic field and the sun, along with a landmark operator model grounded in physical landmarks [131]. The bat algorithm, which leverages the echolocation behavior of bats, is effective for solving single- and multi-objective problems within continuous solution spaces [132]. The gray wolf optimizer, inspired by the social hierarchy and hunting strategies of gray wolves, simulates their natural leadership dynamics [133]. An artificial immune system (ARTIS) encompasses attributes of natural immune systems such as diversity, distributed computation, error tolerance, dynamic learning and adaptation, and self-monitoring, functioning as a robust framework for distributed adaptive systems across various domains [134]. The fruit fly optimization algorithm (FOA), based on the foraging behavior of fruit flies, exploits their keen sense of smell and vision to locate superior food sources and gathering spots [135]. Glow-worm swarm optimization, inspired by glow-worm behavior, is utilized for computing multiple optima of multimodal functions simultaneously [136]. Lastly, invasive weed optimization, inspired by the colonization strategies of weeds, is a numerical stochastic optimization algorithm that mimics the robustness, adaptability, and randomness of invasive weeds using a simple yet effective optimization approach [137].

### 4.6. Gene Feature Extraction

Gene feature extraction refers to the process of extracting meaningful information from genomic data, particularly focusing on the characteristics or attributes of genes. It plays a key role in understanding the biological functions of genes, deciphering genetic mechanisms underlying diseases, and developing computational models for predictive analysis. First, researchers perform original data preprocessing to ensure data quality. Second, what needs to be done is the selection of relevant features; for example, the gene *HER2* is commonly found in breast cancer and stomach cancer. Then, meaningful features are extracted, such as gene expression levels, sequence features, and functional annotations. Finally, based on the extracted features, certain tasks can be achieved, such as classifying cancer genes. Intelligent algorithms are closely related to gene feature extraction, and they are often combined in genomics and bioinformatics research to better understand the function and biological significance of genes.

To prevent some preprocessing that may introduce noise or information loss, ref. [138] proposed a new preprocessing method named mean–standard deviation to solve this problem. In addition, to process overlapping biclusters, Chu [139] introduced a new biclustering algorithm called weight-adjacency-difference-matrix binary biclustering. The results of the *GO enrichment analysis* showed that this method was provided with biological significance using real datasets. Liu [140] proposed the Harris hawks optimization algorithm to accurately select a subset of feature cancer genes. By applying this method to eight published microarray gene expression datasets, the results showed a 100% classification accuracy in gastric cancer, acute lymphoblastic leukemia, and ovarian cancer and an average classification accuracy of 95.33% across a variety of other cancers. Then, ref. [141] improved the Harris hawks optimization algorithm, and the experimental results showed that the classification accuracy of the algorithm was greater than 96.128% for tumors in the colon, nervous system, and lungs, versus 100% for the rest. Compared with seven other algorithms, the experimental results demonstrated the superiority of this algorithm in classification accuracy, fitness value, and the AUC value in feature selection for gene expression data. Inspired by the predatory behavior of humpback whales, Mirjalili [142] proposed a whale optimization algorithm to select the optimal feature gene subset. Compared with other advanced feature selection algorithms, the experimental results showed that the whale optimization algorithm has significant advantages in various evaluation indicators.

### 4.7. Intelligence Communication

Intelligence communication is an emerging, hot field of communication engineering that uses intelligence algorithms to improve the quality of communication. Communication systems can be described simply in three parts: (1) the transmitter, which is responsible for converting information into a signal suitable for transmission, sending it into the transmission-medium encoder, and sending the information to be transmitted; (2) the transmission-medium encoder, in which the transmission medium mainly plays the role of transmitting signals; and (3) the receiver, which receives the signal from the transmission medium and converts it into an intelligible form of information.

Singnal-to-noise ratio estimation is an important task in improving communication quality. Based on deep learning, Xie [143] proposed a new signal-to-noise ratio estimation algorithm using constellation diagrams. This algorithm converted the received signal into a constellation diagram and constructed three deep neural networks to achieve signal-to-noise ratio estimation. The result showed that this algorithm is superior to the traditional algorithm, especially in a low-signal-to-noise-ratio situation.

Cognitive radio is an intelligent wireless communication technology aimed at enhancing the efficiency and reliability of radio spectrum utilization. Traditional wireless communication systems typically allocate fixed spectrum resources to specific users or services, but this allocation method may lead to spectrum wasting and congestion [144]. Cognitive radio dynamically senses, analyzes, and adapts to the radio spectrum environment to achieve the intelligent management and optimization of spectrum resources. False alarm probability and the missed detection probability are usually used to evaluate the performance of spectrum sensing. Consequently, there is a key problem in how to determine a threshold of the false alarm probability and the missed detection probability. There have been many studies related to the selection of threshold and cognitive radio networks [145,146]. Peng [147] proposed two new adaptive threshold algorithms, which had exploited the Markovian behavior of a primary user to reduce the Bayesian cost of a false alarm and missed detection in spectrum sensing. These two algorithms achieved higher detection accuracy for TV white spaces. To improve the performance of the detection time of a cognitive radio network, ref. [148] proposed a fast cooperative energy detection under an accuracy constraints algorithm. The results illustrated that it could achieve a minimal detection time by appropriately choosing the number of secondary users. Differing from the above-mentioned improvements, Liu [149] combined a butterfly network and Clos network and proposed a new topology network to enhance the throughput of the network.

### 4.8. Image Processing

Image processing is a field of study within computer science and engineering that focuses on analyzing, enhancing, and manipulating digital images. With the development of computer science, more and more intelligence algorithms are used in image processing [150,151,152,153]. Due to the outbreak of COVID-19 worldwide, there is a large burden on clinicians. Sun [154] proposed an adaptive-feature-selection, deep-forest algorithm for the classification of the computed tomography chest images of COVID-19 patients. Through the evaluation of the COVID-19 dataset with 1495 patients and 1027 community-acquired pneumonia patients, this algorithm exhibited better performance. To enhance image quality, Wang [138] investigated multifractal theory for texture features through the testing of some textures. The author verified the robustness of the proposed approach in three aspects: noise tolerance, the degree of image blurring, and the compression ratio. From a coding perspective, Li [155] proposed an early merge-mode decision approach for 3D high-efficiency video coding. The proposed approach can effectively decrease the computational cost. In terms of image denoising, Zhu and Chan [156] proposed an approach for restoring images, which is based on mean curvature energy minimization. This approach not only retains all the valid information but also filters out noise.

### 4.9. Other Applications

**Figure 4:**
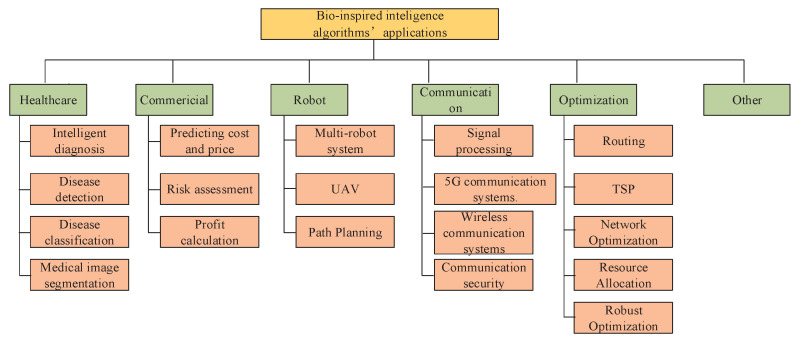
Applications of bio-inspired intelligence algorithms.

Recently, significant research has been directed towards applying intelligence algorithm techniques to address clustering challenges in wireless IoT networks. For instance, the study in [157] proposed a clustering framework specifically for wireless IoT sensor networks. This framework aims to enhance energy efficiency and balance energy consumption using a fitness function optimized via a chicken swarm optimization algorithm. An improved ACO-based UAV path-planning architecture was proposed in [158], which considered obstacles within a controlled area monitored via several radars. The study in [159] proposed a set-based PSO algorithm with adaptive weights to develop an optimal path-planning scheme for a UAV surveillance system. The objective was to minimize energy consumption and enhance the disturbance rejection response of UAVs. Ref. [160] introduced a novel ACO-based fuzzy identification algorithm designed to determine initial consequent parameters and an initial membership function matrix for silted conditions. Zhou [161] presented a new ACO algorithm for continuous domains. This algorithm effectively minimizes total fuel costs by appropriately allocating power demands to generator modules while adhering to various physical and operational constraints. The work in [162] applied the ACO algorithm to develop a new SVM model. The algorithm explored a finite subset of possible values to identify parameters that minimize the generalization error. In the context of flux-cored arc welding, a fusion welding process where a tubular wire electrode is continuously fed into the weld area, the welding input parameters are crucial for determining the quality of the weld joint. Table 1 shows some applications and corresponding papers on bio-inspired intelligence algorithms. Figure 4 depicts when each intelligent algorithm was introduced and some of its main applications.

### 4.10. Discussion and Future Direction

In summary, intelligence algorithms offer versatile solutions across diverse domains, ranging from gene feature extraction to image processing. Whether an intelligence algorithm is bio-inspired or not, both types can tackle complex optimization problems effectively and drive advancements in various fields of research and application. However, there are some challenges, such as local optima traps or overfitting. This has encouraged researchers to explore new optimization strategies. This paper provides some prospective suggestions for future directions.

(1) **Multi-objective optimization:** Extending intelligence algorithms to multi-objective optimization domains is still a hot topic.
(2) **Adaptive and self-learning techniques:** Developing adaptive and self-learning algorithms that automatically adjust parameters and strategies based on problem and environmental changes improves algorithm robustness and adaptability.
(3) **Hybridization and fusion algorithms:** Integrating intelligence algorithms with other optimization methods, such as deep learning and reinforcement learning, to leverage the strengths of various algorithms enhances solution efficiency and accuracy.

## 5. Conclusions

In this paper, we have provided a comprehensive survey of intelligence algorithms. First, we presented an in-depth overview of ZNNs and comprehensively introduced their origin, structure, operation mechanism, model variants, and applications. A novel classification of ZNNs was first proposed, categorizing models into accelerated-convergence ZNNs, noise-tolerance ZNNs, and discrete-time ZNNs, each offering unique advantages in solving time-varying optimization problems. These three types of ZNNs can also be integrated to solve a variety of time-varying optimization algorithms. Concerning applications, the ZNN models were applied in different areas. From different perspectives, some future directions for ZNNs were suggested. Then, we analyzed and outlined other bio-inspired intelligence algorithms, such as GA and PSO, introducing their origins, basic principles, design procedures, and applications. For the rest of the intelligence algorithms, real applications were introduced to emphasize the applicability of intelligent algorithms. In summary, this paper has offered an informative guide for researchers interested in tackling optimization problems using intelligence algorithms.

## Figures and Tables

**Table 1:** Bio-inspired intelligence algorithm and applications.

Algorithm	Applications
Ant colony optimization (ACO)	Scheduling problem [163]
	Routing problem [164]
	Image processing [165]
Particle swarm optimization (PSO)	Neural network training [166]
	Power system [167,168]
	Robots [169]
Gray wolf optimizer (GWO)	Electrical engineering [170]
	Communication [171]
	Mechanical engineering [172]
Whale optimization algorithm (WOA)	Feature selection [173]
	Data cluster [174]
Cuckoo search (CS)	Path optimization [175]
	Scheduling problem [176]
Biogeography-based optimization (BBO)	Healthcare [177]
	Image segmentation [178]
	Feature selection [179]
	Scheduling problem [180]
Flower pollination (FPA)	Electrical power systems [181]
	Engineering optimization [182]
	Wireless and network domain [183]
	Signal and image processing [184,185]
Spiral optimization (SOA)	Scheduling problem [186]
	Path optimization [187]
	Electrical system [188]
Intelligent water drop (IWD)	Wireless sensor network [189]
	Image process [190]
	Path optimization [191]
Cuttlefish optimization (CFO)	Data clustering [192]
	Signal processing [193]

## Data Availability

Some or all of the data and models that support the findings of this study are available from the corresponding author upon request.
